# Sputum Microbiome Based on the Etiology and Severity of Nontuberculous Mycobacterial Pulmonary Disease

**DOI:** 10.3390/jcm14238482

**Published:** 2025-11-29

**Authors:** Junsu Choe, Su-Young Kim, Dae Hun Kim, Byung Woo Jhun

**Affiliations:** Division of Pulmonary and Critical Care Medicine, Department of Medicine, Samsung Medical Center, Sungkyunkwan University School of Medicine, Seoul 06351, Republic of Korea; jefilos@gmail.com (J.C.); suyoung5505@gmail.com (S.-Y.K.)

**Keywords:** nontuberculous mycobacteria, microbiota, severity, *Mycobacterium abscessus*, *Mycobacterium avium*

## Abstract

**Background**: Nontuberculous mycobacterial pulmonary disease (NTM-PD) is a chronic respiratory infection primarily caused by *Mycobacterium avium* complex (MAC) and *Mycobacterium abscessus*. These species differ markedly in antibiotic susceptibility and treatment response, yet the contribution of the respiratory microbiome to this clinical variability remains unclear. To date, however, comparative analyses of microbiome differences between MAC-PD and *M. abscessus*-PD and their associations with disease severity are limited. **Methods**: We conducted microbiome analysis of sputum from 37 patients with NTM-PD. Patients were antibiotic-naïve and classified into MAC-PD (n = 29) and *M. abscessus*-PD (n = 8) groups. Disease severity was determined using radiologic extent on chest computed tomography. Bacterial communities were profiled by 16S rRNA gene sequencing, and differential taxa and predicted functional pathways were analyzed using LEfSe and KEGG orthology databases. **Results**: Distinct microbiome profiles were observed between MAC-PD and *M. abscessus*-PD. Three anaerobic species—*Porphyromonas pasteri*, *Fusobacterium periodonticum*, and *Prevotella nanceiensis*—were significantly enriched in *M. abscessus*-PD (LDA effect size > 3, *p* < 0.05). Functional biomarker analysis revealed significant enrichment of the cobalamin (vitamin B12) biosynthesis pathway in patients with severe disease, while the C19/C18 steroid hormone biosynthesis pathway was enriched in those with mild disease (*p* < 0.05). **Conclusions**: In conclusion, our study demonstrates distinct differences in the respiratory microbiome between MAC-PD and *M. abscessus*-PD and identifies specific functional pathways associated with disease severity in NTM-PD. These findings highlight the potential value of microbial metabolic signatures as biomarkers for disease assessment.

## 1. Introduction

Nontuberculous mycobacterial pulmonary disease (NTM-PD) is a chronic respiratory infection caused by NTM species, and its global disease burden has been increasing steadily [1]. Among NTM species, the *Mycobacterium avium* complex (MAC), which primarily comprises *M. avium* and *M. intracellulare*, is the most frequently isolated pathogen, followed by *Mycobacterium abscessus*, the second most common pathogen in several countries [2,3,4]. Management of NTM-PD generally requires prolonged multidrug therapy based on macrolides for at least several months. However, despite both belonging to the NTM group, MAC and *M. abscessus* exhibit distinct differences in antibiotic susceptibility, treatment response, and clinical prognosis [5,6].

For MAC-PD, ethambutol and rifamycins are typically used in combination with macrolides, whereas the treatment of *M. abscessus*-PD necessitates the use of parenteral antibiotics such as amikacin, imipenem, or cefoxitin. Nevertheless, overall treatment outcomes for NTM-PD remain suboptimal, with *M. abscessus* showing particularly poor responses and a reported cure rate of less than 40%. Furthermore, NTM-PD exhibits substantial heterogeneity in disease progression and severity among patients, resulting in variable clinical outcomes [7]; however, objective biomarkers for reliably assessing disease activity and guiding treatment decisions are currently lacking.

NTM-PD is a multifactorial disease influenced by organism-related factors such as virulence, as well as host immunity and the surrounding microbial environment. Recent evidence indicates that the respiratory microbiome may contribute to the pathogenesis and clinical heterogeneity of NTM-PD, highlighting the potential importance of host–microbe interactions in this disease [8,9,10,11]. Recently, sequencing for conserved bacterial genes, such as the 16S rRNA gene, enables characterization of microbial communities and provides insight into the composition of the respiratory microbiome. However, despite the distinct clinical and microbiological features of MAC and *M. abscessus*, comparative data on differences in their associated respiratory microbiomes are currently lacking. Moreover, no studies have investigated microbial functional pathways that may be linked to disease severity or progression in NTM-PD. To address these knowledge gaps, we analyzed the sputum bacterial microbiomes of 37 antibiotic-naïve patients with MAC-PD or *M. abscessus*-PD.

## 2. Methods

### 2.1. Study Patients and Data Collection

**Figure 1:**
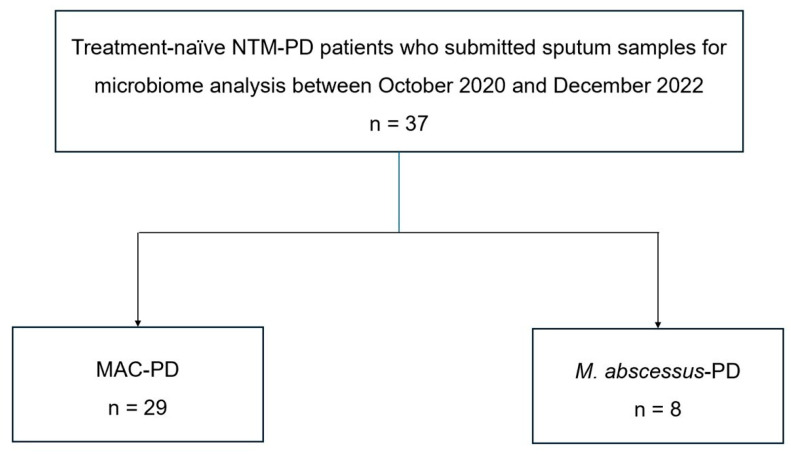
Study participants.

We screened patients with treatment-naïve NTM-PD between October 2020 and December 2022 for microbiome analysis. A total of 37 sputum samples were obtained from 29 patients with MAC-PD and 8 patients with *M. abscessus*-PD (Figure 1). Disease severity was determined based on chest computed tomography. Lesions reflecting NTM-PD activity (consolidation, bronchiectasis, cavity, or bronchiolitis) were assessed by counting the number of affected lung lobes. Each of the five lobes (right upper, right middle, right lower, left upper, and left lower) was assigned one point if involved. Patients with ≥3 affected lobes were classified as the severe group, and those with ≤2 affected lobes as the mild group.

All patients met the diagnostic criteria for NTM-PD [5], and sputum samples were collected and analyzed prior to antibiotic exposure. This study was conducted as a subset of the NTM Registry of Samsung Medical Center (ClinicalTrials.gov identifier: NCT00970801), which investigates the pathophysiology of NTM-PD using clinical data and human-derived materials, including sputum and NTM isolates (IRB no. 2025-01-006). Written informed consent was obtained from all participants.

### 2.2. Sputum Collection and Sequencing

Patients provided at least 3 mL of sputum, distinct from saliva, by coughing deeply into a leak-proof container. Samples were promptly stored at −80 °C and analyzed within three days. DNA was extracted from sputum using the FastDNA^®^ Spin Kit for Soil (MP Biomedicals, Solon, OH, USA). The bacterial 16S rRNA V3–V4 region was amplified, and sequencing libraries were prepared according to the Illumina MiSeq 16S metagenomics protocol. Indexed amplicons were generated using the Nextera XT kit (Illumina, San Diego, CA, USA), purified (QIAquick, Qiagen, Hilden, Germany), and quantified (PicoGreen, Invitrogen, Carlsbad, CA, USA). Library size (400–600 bp) and quality were confirmed by Bioanalyzer 2100 (Agilent, Santa Clara, CA, USA) and agarose gel electrophoresis. Libraries passing quality control were sequenced using the MiSeq Reagent Kit v2 (500 cycles; Illumina) at CJ Bioscience (Seoul, Republic of Korea).

Microbiome profiling was conducted using the 16S-based Microbial Taxonomic Profiling platform in EzBioCloud (PKSSU.4.030). Raw reads were processed through the EzBioCloud pipeline, and taxonomic profiles were normalized by 16S rRNA gene copy number. Relative species abundance was calculated from mapped read counts. Differential taxa between groups were identified using linear discriminant analysis (LDA) effect size (LEfSe), with LDA scores used to estimate effect size (significance threshold *p* < 0.05, LDA effect size value > 3). Hotelling’s *t*-test was used to compare overall bacterial community profiles. Functional prediction was performed using the Kyoto Encyclopedia of Genes and Genomes (KEGG) orthology and pathway databases based on operational taxonomic unit abundance. Clinical variables were compared using the Mann–Whitney U test or Fisher’s exact test, with significance set at *p* < 0.05 [10].

## 3. Results

### 3.1. Baseline Characteristics of Study Patients

The clinical characteristics of the 37 patients with NTM-PD are summarized in Table 1. The median age and body mass index were 59 and 21 kg/m^2^, respectively. Among the patients, 92% were female, and all exhibited nodular bronchiectasis on chest computed tomography, with 14% having cavities larger than 2 cm. Based on the number of affected lobes (1–5 with consolidation, bronchiectasis, cavity, or bronchiolitis), 41% were classified as severe (≥3 lobes), and 59% were in the mild (≤2 lobes) group. The causative organisms were MAC in 29 patients and *M. abscessus* in 8 patients, with no significant clinical differences between the groups.

### 3.2. Differential Sputum Microbiota Between MAC-PD and M. abscessus-PD

We performed LEfSe to compare differences in sputum microbiota composition between the MAC-PD and *M. abscessus*-PD patient groups. At the species level, three taxa were significantly enriched in the *M. abscessus*-PD group compared with the MAC-PD group. *Porphyromonas pasteri* demonstrated the greatest discriminatory power (LDA effect size = 4.20), followed by *Fusobacterium periodonticum* (LDA effect size = 3.85) and *Prevotella nanceiensis* (LDA effect size = 3.66). All these species exhibited significantly higher relative abundance in sputum specimens from patients with *M. abscessus*-PD compared to those with MAC-PD (all *p* < 0.05), indicating a distinct bacterial community structure associated with *M. abscessus* infection.

### 3.3. Microbial Metabolic Pathways Associated with Disease Severity of NTM-PD

We further performed LEfSe analysis to identify functional biomarkers associated with disease severity, classified according to the extent of radiologic involvement on chest computed tomography. Functional pathway prediction was performed using KEGG orthology, and differential module enrichment between groups was evaluated. Among the KEGG modules, cobalamin (vitamin B12) biosynthesis (cobinamide → cobalamin) was significantly enriched in the severe group, suggesting increased microbial metabolic activity associated with cobalamin production (*p* < 0.05). In contrast, the C19/C18 steroid hormone biosynthesis pathway (pregnenolone → androstenedione → estrone) was significantly enriched in the mild group (*p* < 0.05). These differentially enriched modules were mapped to their corresponding KEGG orthologs and metabolic pathways, highlighting distinct functional characteristics of the sputum microbiome between severity groups (Supplementary Figure S1, Supplementary Table S1).

## 4. Discussion

A notable finding of our study was the predominance of specific bacterial species in the sputum samples of patients with *M. abscessus*-PD, in contrast to those with MAC–PD. In particular, *P. pasteri* and *P. nanceiensis*—obligate anaerobic bacteria commonly inhabiting human mucosal surfaces—were more frequently detected in the *M. abscessus*-PD group [12]. These species have previously been implicated in disease-related microbial dysbiosis. For instance, a study employing 16S rRNA gene sequencing to analyze the sputum microbiota of 70 patients with cystic fibrosis demonstrated that the presence of *P. pasteri* and *P. nanceiensis* was associated with a greater annual decline in lung function, suggesting a potential pathogenic or synergistic role in chronic airway disease progression [13]. Meanwhile, *F. periodonticum*, another anaerobic species identified in our cohort, has also been associated with alterations in the respiratory microbiota under pathological conditions. A shotgun metagenomic analysis of sputum samples from 101 patients enrolled in the European Bronchiectasis Registry revealed that the relative abundance of *F. periodonticum* was significantly lower in individuals with bronchiectasis compared with healthy non-smoking controls, suggesting that this species may play a context-dependent role within the airway microbiome [14]. Nevertheless, it remains unclear whether variations in these taxa influence the pathogenesis, host immune response, or clinical outcomes of NTM–PD. Given the distinct clinical characteristics and treatment responses of MAC–PD and *M. abscessus*-PD, further microbiome research is warranted. In particular, longitudinal studies integrating microbial profiling with host immunologic and clinical parameters are needed to clarify the contribution of these taxa to disease progression.

Interestingly, our functional biomarker analysis revealed a marked enrichment of the genetic module associated with cobalamin (vitamin B_12_) biosynthesis in sputum samples from patients in the severe disease group, who also exhibited more extensive radiologic lesions on chest computed tomography. Cobalamin is a structurally complex molecule that functions as an enzymatic cofactor and plays a regulatory role in gene expression. Although *Mycobacterium tuberculosis* is incapable of de novo cobalamin synthesis, recent studies have shown that certain NTM species, including *Mycobacterium smegmatis*, retain the genetic capacity to synthesize cobalamin and may utilize it to support metabolic adaptation and survival under environmental conditions [15,16]. These observations raise the intriguing possibility that cobalamin or its biosynthetic intermediates could serve as potential biomarkers reflecting disease activity or microbial metabolic status in NTM-PD.

In contrast, the genetic module associated with estrone biosynthesis was significantly elevated in the mild disease group. Estrogen, a class of steroid hormones that includes estradiol—the most biologically active form in premenopausal women—and estrone—a less potent form that predominates after menopause—is thought to exert immunomodulatory effects on respiratory diseases. However, the role of estrogen signaling in NTM-PD remains unclear and somewhat controversial. For example, one study reported significantly lower serum estradiol levels in patients with MAC-PD compared with healthy controls [17]. Whereas another investigation found no significant difference in serum estrone levels between patients and controls [18]. These conflicting findings highlight a gap in current understanding and indicate that the interplay between hormones, host immunity, and microbial factors in NTM-PD may be more complex than previously recognized.

Our study has several limitations. First, our study included a relatively small number of samples analyzed. This limitation may have influenced the interpretation of bacterial distributions in sputum from patients with MAC-PD and *M. abscessus*-PD. For example, in our study, although *Enterobacteriaceae* at the genus level showed a slightly higher relative abundance in the MAC-PD group compared with the *M. abscessus*-PD group, the LDA effect size was below 2, indicating low statistical significance. Thus, considering the small sample size, taxa with low LDA effect sizes were excluded from the results. Second, we did not perform NTM species-specific functional biomarker analyses. However, specific pathway alterations or differences in metabolism associated with individual NTM species have not yet been elucidated. Therefore, in this study, we compared functional pathways according to disease severity irrespective of the NTM species. Further studies with larger sample sizes are required to address this issue. Third, it is well known that the presence of cavitary lesions reflects the severity of NTM-PD. However, among the 37 patients included in our study, only four had cavities. Therefore, we considered it limited and not appropriate to compare microbiome differences solely based on the presence or absence of cavities. Instead, we evaluated disease severity according to the overall extent of pulmonary involvement. For reference, our research group previously analyzed microbiomes using surgical specimens from patients with advanced cavitary lung lesions [19]. Nevertheless, studies investigating microbiome changes in relation to cavity formation or disease severity in NTM-PD remain scarce. Lastly, our current study by itself does not have sufficient impact to change clinical practice at this stage. Considering that the pathophysiology of NTM-PD, including disease-related microbial environments and the lack of biomarkers that reflect disease severity, remains unclear, further advanced studies are needed.

In conclusion, our study demonstrates distinct differences in the respiratory microbiome between MAC-PD and *M. abscessus*-PD and identifies specific microbial functional pathways associated with disease severity in NTM-PD. These findings suggest that microbial metabolic signatures, including cobalamin and estrone biosynthesis pathways, may serve as potential biomarkers for assessing disease activity and informing clinical management.

## Figures and Tables

**Table 1:** Clinical characteristics of the study patients.

Characteristics	Total (n = 37)	MAC (n = 29)	*M. abscessus* (n = 8)	*p*-Value
Age, yrs	59 (53–66)	60 (54–67)	53 (50–57)	0.119
Sex, female	34 (92)	26 (90)	8 (100)	>0.999
Body mass index, kg/m^2^	21 (20–23)	22 (20–23)	21 (21–22)	0.915
Positive AFB smear	7 (19)	4 (14)	3 (38)	0.156
Number of lobes involved by bronchiectasis ^¶^	2 (1–3)	2 (1–2)	2 (2–3)	0.435
Cavitary lesion on CT	5 (14)	4 (14)	1 (13)	>0.999
Severe group ^#^	15 (41)	11 (38)	4 (50)	0.690

Data are presented as numbers (percentages) or medians (interquartile ranges). AFB, acid-fast bacilli; CT, computed tomography; MAC, *Mycobacterium avium* complex. ^¶^ All exhibited the nodular bronchiectasis form on chest CT scan. ^#^ Patients were further classified into severe (≥3 lobes) and mild (≤2 lobes) groups based on the number of affected lobes observed on chest CT (minimum of 1 lobe-maximum of 5 lobes with consolidation, bronchiectasis, cavity, or bronchiolitis).

## Data Availability

The raw data were registered in the Sequence Read Archive data (Bioproject) and are available under the accession number PRJNA1151138 (Release date: 30 September 2025).

## Supplementary Materials

The following supporting information can be downloaded at: https://www.mdpi.com/article/10.3390/jcm14238482/s1. Figure S1: Differences in the Kyoto Encyclopedia of Genes and Genomes (KEGG) pathway profiles between groups determined using LEfSe analysis (Logarithmic LDA score > 1.5, *p*-value < 0.05). Module enrichment for KEGG cellular processes in severe and mild disease groups. Table S1: The mean relative abundances of KEGG modules and KEGG orthologs involved in the pathways identified using LEfSe analysis.

## References

[1] Prevots D.R., Marshall J.E., Wagner D., Morimoto K. (2023). Global epidemiology of nontuberculous mycobacterial pulmonary disease: A review. Clin. Chest Med..

[2] Prevots D.R., Marras T.K. (2015). Epidemiology of human pulmonary infection with nontuberculous mycobacteria: A review. Clin. Chest Med..

[3] Hoefsloot W., van Ingen J., Andrejak C., Angeby K., Bauriaud R., Bemer P., Beylis N., Boeree M.J., Cacho J., Chihota V. (2013). The geographic diversity of nontuberculous mycobacteria isolated from pulmonary samples: An NTM-NET collaborative study. Eur. Respir. J..

[4] Jeon D. (2019). Infection source and epidemiology of nontuberculous mycobacterial lung disease. Tuberc. Respir. Dis..

[5] Griffith D.E., Aksamit T., Brown-Elliott B.A., Catanzaro A., Daley C., Gordin F., Holland S.M., Horsburgh R., Huitt G., Iademarco M.F. (2007). An official ATS/IDSA statement: Diagnosis, treatment, and prevention of nontuberculous mycobacterial diseases. Am. J. Respir. Crit. Care Med..

[6] Daley C.L., Iaccarino J.M., Lange C., Cambau E., Wallace R.J., Andrejak C., Böttger E.C., Brozek J., Griffith D.E., Guglielmetti L. (2020). Treatment of nontuberculous mycobacterial pulmonary disease: An official ATS/ERS/ESCMID/IDSA clinical practice guideline. Eur. Respir. J..

[7] Kim B.G., Jhun B.W., Kim H., Kwon O.J. (2022). Treatment outcomes of *Mycobacterium avium* complex pulmonary disease according to disease severity. Sci. Rep..

[8] Huang H.L., Lin C.H., Lee M.R., Huang W.C., Sheu C.C., Cheng M.H., Lu P.L., Huang C.H., Yeh Y.T., Yang J.M. (2024). Sputum bacterial microbiota signature as a surrogate for predicting disease progression of nontuberculous mycobacterial lung disease. Int. J. Infect. Dis..

[9] Sulaiman I., Wu B.G., Li Y., Scott A.S., Malecha P., Scaglione B., Wang J., Basavaraj A., Chung S., Bantis K. (2018). Evaluation of the airway microbiome in nontuberculous mycobacteria disease. Eur. Respir. J..

[10] Song M.J., Kim D.H., Kim S.Y., Kang N., Jhun B.W. (2024). Comparison of the sputum microbiome between patients with stable nontuberculous mycobacterial pulmonary disease and patients requiring treatment. BMC Microbiol..

[11] Philley J.V., Kannan A., Olusola P., McGaha P., Singh K.P., Samten B., Griffith D.E., Dasgupta S. (2019). Microbiome diversity in sputum of nontuberculous mycobacteria infected women with a history of breast cancer. Cell. Physiol. Biochem..

[12] Guilloux C.A., Lamoureux C., Beauruelle C., Héry-Arnaud G. (2021). Porphyromonas: A neglected potential key genus in human microbiomes. Anaerobe.

[13] Webb K., Zain N.M.M., Stewart I., Fogarty A., Nash E.F., Whitehouse J.L., Smyth A.R., Lilley A.K., Knox A., Williams P. (2022). *Porphyromonas pasteri* and *Prevotella nanceiensis* in the sputum microbiota are associated with increased decline in lung function in individuals with cystic fibrosis. J. Med. Microbiol..

[14] Rosenboom I., Thavarasa A., Richardson H., Long M.B., Wiehlmann L., Davenport C.F., Shoemark A., Chalmers J.D., Tümmler B. (2024). Sputum metagenomics of people with bronchiectasis. ERJ Open Res..

[15] Minias A., Gąsior F., Brzostek A., Jagielski T., Dziadek J. (2021). Cobalamin is present in cells of non-tuberculous mycobacteria, but not in *Mycobacterium tuberculosis*. Sci. Rep..

[16] Kipkorir T., Mashabela G.T., de Wet T.J., Koch A., Dawes S.S., Wiesner L., Mizrahi V., Warner D.F. (2021). De novo cobalamin biosynthesis, transport, and assimilation and cobalamin-mediated regulation of methionine biosynthesis in *Mycobacterium smegmatis*. J. Bacteriol..

[17] Uwamino Y., Nishimura T., Sato Y., Tamizu E., Asakura T., Uno S., Mori M., Fujiwara H., Ishii M., Kawabe H. (2019). Low serum estradiol levels are related to *Mycobacterium avium* complex lung disease: A cross-sectional study. BMC Infect. Dis..

[18] Danley J., Kwait R., Peterson D.D., Sendecki J., Vaughn B., Nakisbendi K., Sawicki J., Lande L. (2014). Normal estrogen, but low dehydroepiandrosterone levels, in women with pulmonary *Mycobacterium avium* complex. A preliminary study. Ann. Am. Thorac. Soc..

[19] Kim B.G., Kang N., Kim S.Y., Kim D.H., Kim H., Kwon O.J., Huh H.J., Lee N.Y., Jhun B.W. (2023). The lung microbiota in nontuberculous mycobacterial pulmonary disease. PLoS ONE.

