# Supplementary material for: Sputum Microbiome Based on the Etiology and Severity of Nontuberculous Mycobacterial Pulmonary Disease
Source: J Clin Med. 2025 Nov 29;14(23):8482. doi: 10.3390/jcm14238482 (PMC12692926; doi:10.3390/jcm14238482)
Supplement: Supplementary file 1 [file jcm-14-08482-s001.zip › Microbiome NTM_Supplement_JCM submission.pdf]

Supplementary Figure S1. Differences in the Kyoto Encyclopedia of Genes and Genomes (KEGG) pathway profiles between groups determined using LEfSe analysis (Logarithmic LDA score >1.5, p-value < 0.05). Module enrichment for KEGG cellular processes in severe and mild disease groups.

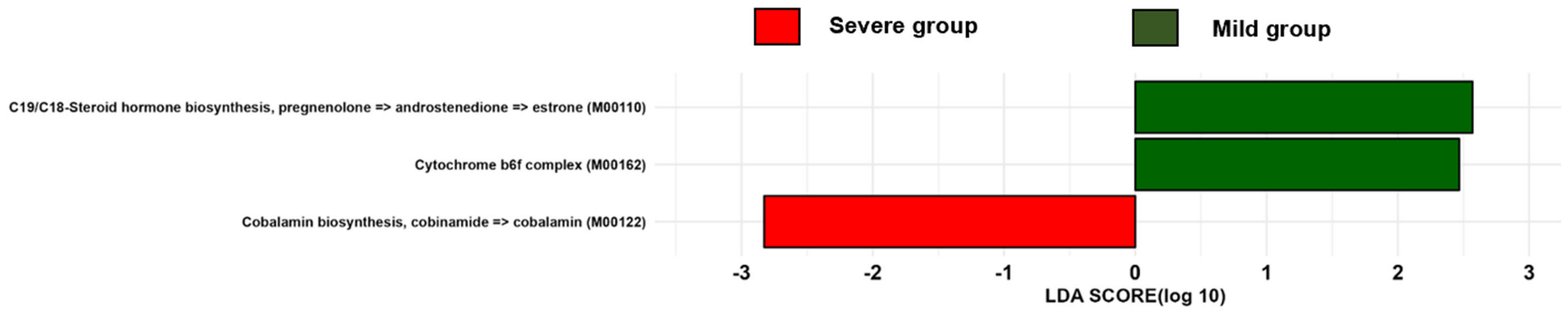

Supplementary Table S1. The mean relative abundances of KEGG modules and KEGG orthologs involved in the pathways identified using LEfSe analysis.

KEGG module	Definition	Sever group	Mild group	p-value	Orthology	Definition	Involved pathways	Sever group	Mild group	p-value
M00122	Cobalamin biosynthesis, cobinamide => cobalamin	0.507272723	0.379752763	0.038209	K19221	cob(I)alamin adenosyltransferase	ko00860, ko01100	0.036975597	0.024572883	0.005368
M00110	C19/C18-Steroid hormone biosynthesis, pregnenolone => androstenedione => estrone	0.095286352	0.152882444	0.038209	K07434	aromatase	ko00140, ko01100, ko04913	0.051841126	0.083832919	0.038209
M00162	Cytochrome b6f complex	0.092895056	0.141978537	0.047726	-	-	-	-	-	-
